# Extracellular Matrix Features Discriminate Aggressive HER2-Positive Breast Cancer Patients Who Benefit from Trastuzumab Treatment

**DOI:** 10.3390/cells9020434

**Published:** 2020-02-13

**Authors:** Ilona Rybinska, Marco Sandri, Francesca Bianchi, Rosaria Orlandi, Loris De Cecco, Patrizia Gasparini, Manuela Campiglio, Biagio Paolini, Lucia Sfondrini, Elda Tagliabue, Tiziana Triulzi

**Affiliations:** 1 Molecular Targeting Unit, Department of Research, Fondazione IRCCS Istituto Nazionale dei Tumori, 20133 Milan, Italy; ilona.rybinska@istitutotumori.mi.it (I.R.); francesca.bianchi@istitutotumori.mi.it (F.B.); rosaria.orlandi@istitutotumori.mi.it (R.O.); manuela.campiglio@me.com (M.C.); tiziana.triulzi@istitutotumori.mi.it (T.T.); 2 Data Methods and Systems Statistical Laboratory, University of Brescia, 25121 Brescia, Italy; sandri.marco@gmail.com; 3 Platform of Integrated Biology, Department of Applied Research and Technology Development, Fondazione IRCCS Istituto Nazionale dei Tumori, 20133 Milan, Italy; Loris.DeCecco@istitutotumori.mi.it; 4 Genomic Unit, Department of Research, Fondazione IRCCS Istituto Nazionale dei Tumori, 20133 Milan, Italy; patrizia.gasparini@istitutotumori.mi.it; 5 Anatomic Pathology A Unit, Department of Pathology, Fondazione IRCCS Istituto Nazionale dei Tumori, 20133 Milan, Italy; biagio.paolini@istitutotumori.mi.it; 6 Dipartimento di Scienze Biomediche per la Salute, Università degli Studi di Milano, 20133 Milan, Italy; lucia.sfondrini@unimi.it

**Keywords:** extracellular matrix, HER2 positive breast cancer, gene expression, ECM3, collagen, trastuzumab

## Abstract

We previously identified an extracellular matrix (ECM) gene expression pattern in breast cancer (BC), called ECM3, characterized by a high expression of genes encoding structural ECM proteins. Since ECM is reportedly implicated in response to therapy of BCs, the aim of this work is to investigate the prognostic and predictive value of ECM3 molecular classification in HER2-positive BCs. ECM3 resulted in a robust cluster that identified a subset of 25–37% of HER2-positive tumors with molecular aggressive features. ECM3 was significantly associated with worse prognosis in two datasets of HER2-positive BCs untreated with adjuvant therapy. Analyses carried out on two of our cohorts of patients treated or not with adjuvant trastuzumab showed association of ECM3 with worse prognosis only in patients not treated with trastuzumab. Moreover, investigating a dataset that includes gene profile data of tumors treated with neoadjuvant trastuzumab plus chemotherapy or chemotherapy alone, ECM3 was associated with increased pathological complete response if treated with trastuzumab. In the in vivo experiments, increased diffusion and trastuzumab activity were found in tumors derived from injection of HER2-positive cells with Matrigel that creates an ECM-rich tumor environment. Taken together, these results indicate that HER2-positive BCs classified as ECM3 have an aggressive phenotype but they are sensitive to trastuzumab treatment.

## 1. Introduction

The extracellular matrix (ECM) of a tumor is composed of a complex mixture of proteins that, other than providing structural support to cells and tissues, influences tumor progression and response to therapy [1,2]. Differences in ECM-associated gene expression are able to describe the biological and clinical heterogeneity of breast cancer (BC). At Fondazione IRCCS Istituto Nazionale dei Tumori (INT), we identified an extracellular matrix gene expression pattern (ECM3) in ~40% of BCs that is able to classify a group of tumors independent of their molecular intrinsic subtype and is associated with poor prognosis in patients with undifferentiated grade III tumors [3,4]. These tumors are characterized by overexpression of a robust cluster of genes mainly encoding structural ECM proteins and by overexpression of pathways related to epithelial to mesenchymal transition (EMT). The induction of EMT in tumor cells is driven by the interplay between myeloid-derived suppressor cells (MDSCs) and the ECM [5].

Breast cancer is the most frequent female type of cancer and the leading cause of cancer-related mortality worldwide. The American Cancer Society estimated 250,000 new BC cases and ~40,000 deaths from this disease in 2019 [6]. The HER2 gene is amplified and/or overexpressed in approximately 20% of BCs [7], and has been associated with tumor progression and poor prognosis [8]. Trastuzumab, a recombinant humanized monoclonal antibody that binds to the extracellular domain of HER2, has changed the natural history of these tumors, improving patient survival [9]. However, this antibody, administered per currently approved protocols, is effective in ~50% of patients with this type of tumor. No predictive biomarkers are clinically available [10], even if pathological and molecular characterization of tumor tissues has identified tumor-intrinsic features (ER, PAM50 and TRAR) [10,11,12] and immune features [13] as predictive of trastuzumab efficacy.

Since ECM is reportedly implicated in response to therapy of BCs [14] and ECM3 contained 35–40% of HER2-positive BCs [4], herein, we tested the prognostic and predictive value of ECM3 molecular classification in HER2-positive BCs.

## 2. Materials and Methods

### 2.1. Patients

The 44 tumors of the FIRB cohort were obtained from BC patients treated with adjuvant chemotherapy between 1981 and 2002, after selection based on HER2 positivity by immunohistochemistry (IHC) (3+ or 2+) and fluorescence in situ hybridization (FISH) (only tested in IHC 2+) as potential targets of trastuzumab treatment. All selected samples contained at least 70% tumor cells.

All procedures were in accordance with the Helsinki Declaration. Biospecimens used for research consisted of leftover material of samples collected during standard surgical and medical approaches at INT. Samples were donated by patients to the Institutional BioBank for research purposes, and aliquots were allocated to this study after approval by the Institutional Review Board and a specific request to the Independent Ethical Committee of the institute.

### 2.2. Immunohistochemistry and Fluorescence In Situ Hybridization

HER2, ER and PGR positivity of cases of the FIRB cohort were re-evaluated (HER2: A0485; ER: clone 1D5, M7047; PGR: clone PgR636, M3569, Dako, Santa Clara, CA, USA) on formalin-fixed, paraffin-embedded (FFPE) tissue sections. The immunoreaction was developed using the streptavidin–biotin–peroxidase technique, followed by counterstaining with Carazzi hematoxylin. HER2 positivity was defined as 3+ overexpression in more than 10% of tumor cells by IHC or 2+ overexpression and HER2 amplification ratio of at least 2.2 by FISH. Tumors were considered ER- or PGR-positive if at least 10% of cells showed immunoreactivity.

All HER2 2+ cases were evaluated by FISH using the PathVysion HER-2DNA Probe kit (Abbott, Chicago, IL, USA) according to the manufacturer’s recommendations, as previously described [15]. Briefly, 2 µm FFPE sections were deparaffined in Hybrite (Abbott) and rehydrated. After pepsin treatment (0.01 N HCl + 0.4% pepsin) at 37 °C for 6 min, samples were denatured at 85 °C for 1 min and hybridized with probes overnight at 37 °C. Samples were stained with 4′,6-diamidino-2-phenylindole, cover-slipped and analyzed with a Zeiss Axioscop 2 microscope (Carl Zeiss, Oberkochen, Germany).

### 2.3. RNA Isolation and cDNA Microarray Techniques

Total RNA was extracted from snap-frozen samples using Trizol (Thermo Fisher Scientific, Waltham, MA, USA) following the manufacturer’s instructions. Integrity of the RNA was assessed by agarose gel electrophoresis after DNase I and clean-up treatment with RNeasy mini kit (Qiagen, Hilden, Germany). All processed tumor specimens contained at least 70% cancer cells. A radiolabeled tracer was added during the cDNA synthesis and a small amount of the cDNA was run on an alkaline gel (1% agarose, 50 mM NaOH, 1 mM EDTA). Only cDNAs showing a similar core size of 1–1.5 k nucleotides were used for subsequent hybridization. The target cDNAs were synthesized from total RNA and directly labeled with Cy3-dCTP (reference RNA) or Cy5-dCTP (sample RNA) (GE Healthcare, Buckinghamshire, UK) and indirectly with 3DNA Submicro Expression Array Detection kit (Genisphere, Hatfield, PA, USA). Total RNA was reverse transcribed using 5′ end modified oligo-dT primers containing the specific Cy3 or Cy5 3DNA capture sequences. The ^32^P-labeled cDNAs with an estimated activity of about 100,000 counts per minute were annealed with 1.5 µL of the specific DNA capture reagent for 16 h at 41 °C. Hybridization was carried out in a hybridization station (Genomic Solutions, Cridersville, GE, USA) and slides were scanned using the GenePix 4000A microarray scanner (Molecular Devices, Sunnyvale, CA, USA).

### 2.4. Data Analysis

Dataset filtering and Lowess normalization were performed using J-Express (Molmine, Hafrsfjord, Norway) as described [16]. Bioinformatic analyses were performed using R [17], version 2.15, and BioConductor [18], release 2.10. Gene-set enrichment analyses were performed using gene set enrichment analysis GSEA v5.0 [19]. Genes represented by more than one probe were collapsed to the probe with the maximum value using the Collapse Dataset tool. Gene-set permutation was applied 1000 times and gene-set enrichment was considered significant at FDR < 25%.

### 2.5. External Datasets and Signatures

ECM3 was calculated in HER2-positive patients of the following public datasets: NKI (Netherlands Cancer Institute) (untreated) [20]; ii) EMC (untreated) [21]; GSE50948 (NOAH trial, treated with neo-adjuvant chemotherapy alone or chemotherapy and trastuzumab) [22]. Microarray data were analyzed starting from processed data retrieved from GEO. Gene expression data of the GHEA cohort (GSE55348), containing tumors treated with adjuvant trastuzumab-based therapy in our institute, were also used [23]. HER2-overexpressing tumors in EMC and NKI datasets were selected based on mRNA levels of genes in the 17q12 amplicon reported to be overexpressed in HER2-positive tumors, as described [24]. In detail, cluster analysis was performed with genes belonging to the *ERBB2* amplicon after performing median centering of the amplicon genes using Cluster 3.0 and TreeView softwares. Tumors in the cluster with overexpression of these genes were considered amplified.

ECM3 was identified using the Large Average Submatricies (LAS) biclustering method [25]; compactness and separation of cluster partitions were evaluated using the Dunn index and silhouette width [26]; connectedness of clusters was quantified using the connectivity measure [26], as previously described [4].

The research-based PAM50 subtype predictor was applied to the GHEA and NOAH datasets using the publicly available algorithm as described [22], after performing median centering of the PAM50 genes. Immune metagenes were determined based on the method of Rody et al. [27]. The average log-transformed expression of the genes that belonged to each metagene was calculated.

### 2.6. In Vivo Study

Experimental protocols were approved by the Ethics Committee for Animal Experimentation of Fondazione IRCCS-INT. Care and use of the animals were in accordance with institutional guidelines. Six- to eight-week-old CD1 athymic female mice were purchased from Charles River. Mice (4/group), housed 4 per cage (1 cage for group) with food and water available ad libitum, were injected subcutaneously with 1 × 10^6^ SKOV3 cells in PBS. HER2-positive SKOV3 cells purchased from the American Type Culture Collection (ATCC) were cultured in RPMI 1610 medium (Euroclone, Pero, Italy) supplemented with 10% fetal bovine serum (FBS) (Thermo Fisher Scientific) in a humidified chamber (95% air, 5% CO_2_) at 37 °C. Cells were authenticated using the Short Tandem Repeat Profiling method in our institute’s facility. To generate an extracellular matrix-rich model, cells were embedded in 100 μL of Matrigel and injected in athymic mice. To assess trastuzumab-mediated therapeutic effects, trastuzumab was administered 4 mg/kg i.p. twice a week starting one week after cell injection. Tumors were calibrated once a week and tumor volume was calculated as 0.5 × d_1_^2^ × d_2_, where d_1_ and d_2_ are the smaller and largest diameters, respectively.

To quantify the amount of trastuzumab that reached the tumor tissue, mice bearing 0.2 cm^3^ tumors were treated with 10 mg/kg ^131^I-Trastuzumab and analyzed for antibody radiolocalization 48 h after drug injection. Radiolabeling was carried out using the Chizzonite method for indirect protein iodination with IODO-GEN^®^ pre-coated tubes following the manufacturer’s instructions (Thermo Fisher Scientific). The obtained radiolabeled antibody was diluted with saline solution to a final concentration of ~6 MBq/mL and injected at 1.3–1.5 MBq/0.25 mL/mouse. Radioactivity was quantified in tumors and in muscle as reference with COBRA II Auto-Gamma counter (Packard, PerkinElmer, Waltham, MA, USA).

Trastuzumab was also quantified 24 h after injection by immunofluorescence. Tumors were removed, fixed in 4% paraformaldehyde and OCT, frozen in liquid nitrogen, sectioned (5-µm), and processed for immunofluorescence (IF) labeling. Slides were saturated with 3% BSA and incubated with mouse Ab anti-mouse collagen I-III (NB600-450 and NB600-451, Novus Biologicals, Centennial, CO, USA) and incubated with a specific Alexafluor-conjugated (Thermo Fisher Scientific) secondary antibody. Trastuzumab was visualized using Alexafluor-conjugated anti human secondary antibody. Nuclei were visualized by Sybr-green. Coverslips were mounted on glass slides using Prolong (Calbiochem, San Diego, CA, USA) and examined with a confocal microscope (Microradiance 2000, BioRad, Hercules, CA, USA) equipped with Argon (488 nm), Green HeNe (543 nm) and Red diode (633 nm) lasers. Images were obtained using a ×60 oil immersion lens (512 × 512 or 1024 × 1024 pixels) and analyzed using Image-Pro Plus v. 7.0.1 (MediaCybenetics, Rockville, MD, USA) software.

### 2.7. Statistical Analysis

Relationships between categorical variables were assessed using Fisher’s exact test. Two-tailed Student’s *t*-test was used to compare mean values of two independent groups.

Disease-free survival (DFS) was defined as the time elapsed from the date of surgery to the date of the first event. Overall survival (OS) was defined as the time elapsed from the date of surgery to the date of death from any cause or the date of last follow-up. Kaplan–Meier survival plots and exact log-rank tests were used to assess differences in DFS and OS between two groups. Adjusted hazard ratios (HRs) of prognostic factors together with 95% confidence intervals (CIs) were estimated by fitting multivariable Cox survival models. All the analyses were conducted using SAS software (SAS Institute Inc., Cary, NC, USA). Two-sided *p*-values < 0.05 were considered statistically significant.

## 3. Results

### 3.1. Identification of ECM3 HER2-Positive Breast Carcinomas

To test the relevance of our previously described signature ECM3 [4] in HER2-positive BCs, we studied the expression profile of ECM-related genes by LAS method in 5 cohorts of primary HER2-positive BCs. As already described for consecutive BCs, ECM3 was detectable in every dataset analyzed. In total, 25% to 37% of the cases were classified as ECM3 (Table 1). The average silhouette of ECM3 clusters in the 5 datasets ranged from 0.21 to 0.34, the Dunn indices ranged from 0.45 to 0.59 and connectivity ranged from 5.4 to 12.4, indicating a highly significant partition of ECM3 subset also among HER2-positive BCs.

Contrary to what we previously observed in BC cohorts containing tumors of all intrinsic subtypes [4], in which ECM3 tumors were mainly ER positive and grade I-II, ECM3 was not significantly associated with any clinico-pathological variable in all the datasets analyzed (Table 1).

**Figure 1:**
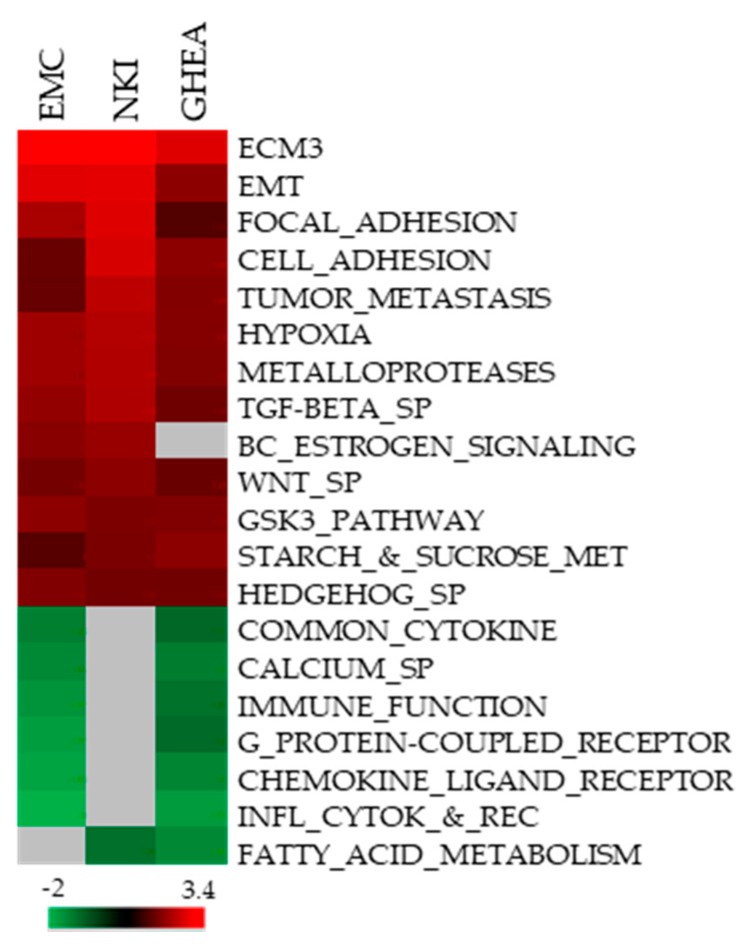
Molecular characteristics of HER2-positive breast cancers (BCs) according to ECM classification. Heatmap shows the normalized enrichment scores (NES) of gene sets significantly enriched at FDR < 25% in ECM3 tumors in at least two datasets, by GSEA analysis. Grey boxes indicated no enrichment.

To explore the possibility of providing biologically meaningful insights to this classification in HER2-positive tumors, we used GSEA. Comparison of gene expression in ECM3 vs. non-ECM3 tumors revealed significant enrichment for pathways we previously found associated with ECM3, such as EMT, cell and focal adhesion, TGF-beta pathway, tumor metastasis and hypoxia (Figure 1), confirming the biological characteristics of ECM3 tumors also among HER2-positive tumors. Genes related to inflammation were found enriched in non-ECM3 tumors (Figure 1), as previously observed [4]. Nevertheless, this enrichment was not observed in all analyzed datasets.

### 3.2. ECM3 in HER2-Positive BCs Not Treated with Trastuzumab

**Figure 2:**
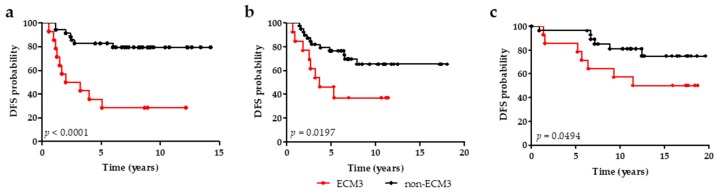
ECM3 prognostic significance in HER2-positive BC patients. (**a**,**b**) Association between ECM3 (red line) and non-ECM3 (black line) and disease-free survival (DFS) in untreated HER2-positive BC patients of the EMC (**a**, *n* = 49) and NKI (**b**, *n* = 52) datasets. (**c**) Association between ECM3 and non-ECM3 and DFS in HER2-positive BC patients (FIRB cohort) treated with adjuvant chemotherapy without trastuzumab. *p*-values by log-rank test.

To test the prognostic significance of ECM classification in HER2-positive tumors the performance of ECM3 was investigated in 49 and 52 untreated HER2-positive tumors from EMC and NKI datasets, respectively. Univariate analysis indicated worse DFS for ECM3 tumors in both datasets (Figure 2 and Table S1). Moreover, ECM3 resulted an independent prognostic factor of worse DFS in multivariate analysis with estrogen receptor (ER) in both datasets (HR = 5.50, 95% CI = 2.07–14.6 and HR = 2.57, 95% CI = 1.06–6.19, respectively) (Table 2). ECM3 remained the variable most associated with DFS probability in the NKI dataset in multivariate analysis with other available clinico-pathological variables (Tables S1 and S2). OS in NKI patients with ECM3 tumors was also significantly worse (Table S3).

We next evaluated prognosis of patients with ECM3 tumors treated with adjuvant chemotherapy. A total of 44 HER2-positive cases (35 samples 3+ and 9 samples 2+/FISH positive) treated with chemotherapy in our institute before the introduction of trastuzumab in the clinical practice were selected. The Kaplan–Meier curve showed a worse prognosis for patients with ECM3 tumors (*p* = 0.0494, Figure 2). ECM3 was the variable more associated with DFS and OS both in univariate (Table S1) and multivariate analyses (Table 2 and Table S3), indicating that ECM3 cases have a worse prognosis even if treated with chemotherapy.

### 3.3. ECM3 in HER2-Positive BC Treated with Trastuzumab

**Figure 3:**
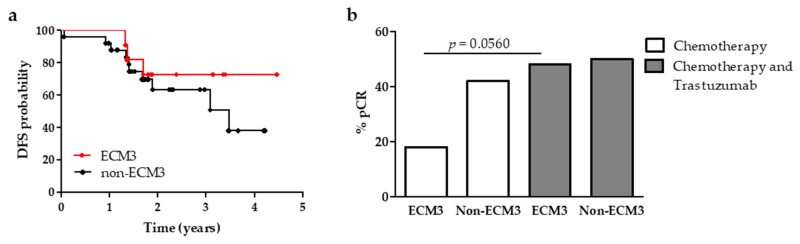
ECM3 predictive significance of trastuzumab benefit. (**a**) Association between ECM3 (red line) and non-ECM3 (black line) and DFS in HER2-positive BC patients treated with adjuvant trastuzumab-based therapy (GHEA cohort, *n* = 36). (**b**) Percentage of pathological complete response (pCR) in ECM3 and non-ECM3 cases according to neoadjuvant treatment (NOAH dataset). *p*-value by Fisher exact test.

To understand whether ECM3 cases could benefit from trastuzumab treatment, we analyzed the ECM signature in a cohort of patients treated in our institute with adjuvant trastuzumab-based therapy. Cases of the GHEA cohort [23] were submitted to LAS biclustering to identify ECM3 tumors. ECM3 was not associated with any clinico-pathological variables (Table 1) and it was not found associated with worse prognosis in these cases, but a trend toward better prognosis was observed (Figure 3), suggesting that ECM3 cases could benefit from trastuzumab treatment. We also investigated whether ECM3 was associated with other predictors of trastuzumab benefit, like PAM50 [22] and TRAR, that were previously developed in the GHEA datasets [23]. No association was found between ECM3 and HER2-enriched or TRAR-low, the two subsets of cases who best benefit from trastuzumab treatment (Figure S1).

Analysis of the tumor immune microenvironment showed higher positivity in CD8+ cells in ECM3 tumors than non-ECM3 tumors (10/11 vs. 9/24, *p* = 0.0041) of the GHEA cohort. This association was confirmed by higher expression of *CD8A* gene in ECM3 than non-ECM3 cases of the FIRB cohort (mean = 0.35 95% CI = 0.15–0.55 vs. mean = 0.08 95% CI = −0.10–0.25, *p* = 0.0452).

To determine whether ECM3 is predictive of trastuzumab response, this signature was tested in cases of the NOAH dataset that were treated with neoadjuvant chemotherapy alone or with chemotherapy and trastuzumab. Also in this dataset no association was found between ECM3 and any clinico-pathological variable (Table 1) and with PAM50 and TRAR (Figure S1). Interestingly, we observed that only 18% of ECM3 cases achieved pathological complete response (pCR) when treated with chemotherapy alone, while treatment with trastuzumab doubled the percentage of pCR (48%, *p* = 0.0560). Non-ECM3 cases respond well both to chemotherapy (42% of pCR) and trastuzumab (50% of pCR, *p* = 0.0948, Figure 3). To more deeply investigate the association between ECM3 and immune signatures in relation to pCR, we calculated the value of the immune metagenes described to be associated with pCR in this dataset [28]. Hemopoietic cell kinase (HCK) metagene and MHC-II metagene, surrogate markers of cells belonging to the monocyte/myeloid lineage and of antigen-presenting cells, respectively, were found to be significantly higher expressed in ECM3 tumors (*p* < 0.001), but not associated with response either in cases of the chemotherapy arm or in those receiving trastuzumab (Figure S2). On the contrary, lymphocyte specific kinase (LCK) and STAT1 metagenes were found significantly more expressed in ECM3 tumors of patients who achieved pCR vs. residual disease (RD) only if treated with trastuzumab (Figure S2), suggesting a possible combination of these markers to improve prediction of trastuzumab benefit.

### 3.4. Extracellular Matrix and Response to Trastuzumab in a Xenograft Model

**Figure 4:**
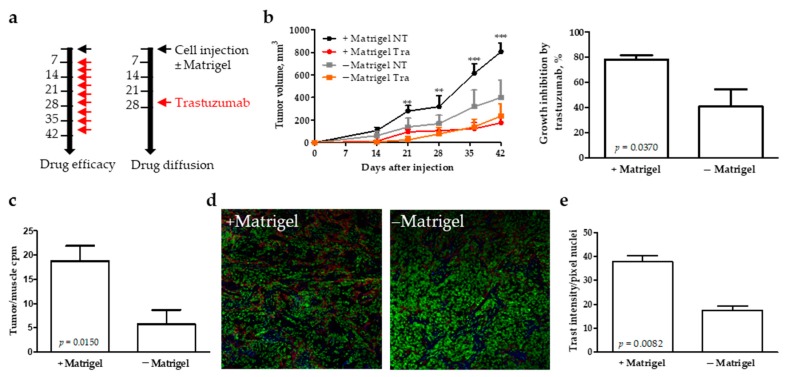
Role of extracellular matrix density in trastuzumab efficacy. (**a**) Scheme of the in vivo experiments. (**b**) Tumor volume and percent tumor growth inhibition by trastuzumab at day 42 in tumors obtained from injection of cells with (+ Matrigel) or without (− Matrigel) Matrigel. Data are mean ± SD (*n* = 4). (**c**) ^131^I-Trastuzumab quantification in tumors obtained from injection of cells in mice with (+ Matrigel) or without (− Matrigel) Matrigel 48 h after treatment with ^131^I-Trastuzumab. Data are mean ± SD of the ratio between radioactivity (count per minute) quantified in the tumor and in the muscle of the same mouse (*n* = 5). (**d**) Representative images of trastuzumab localization evaluated by immunofluorescence in tumors of mice injected with cells embedded (+) or not (−) in Matrigel (red: trastuzumab, green: sybr green, nuclei, blue: collagen). (**e**) Trastuzumab quantification in tumors grown with (+) or without (−) Matrigel 24 h after treatment. Data are mean ± SD (*n* = 4) of the mean fluorescence intensities of red positive pixels normalized on the number of green pixels (nuclei). *p*-values by unpaired *t*-test.

To test whether the presence of high amounts of extracellular matrix overexpressed in ECM3 tumors could impact trastuzumab diffusion and antitumor activity, the HER2-amplified carcinoma cell line SKOV3 was injected in nude mice with or without Matrigel and then treated with trastuzumab (Figure 4a). No sign of toxicity by treatment was observed (Figure S3). While the presence of Matrigel increased the growth rate of these xenografts, trastuzumab-mediated reduction of tumor growth was significantly greater in tumors grown in ECM-rich tumors compared with those grown in the absence of ECM (Figure 4b). Ki67 immunoreactivity of tumor tissues collected at the end of the treatment was not reduced by trastuzumab both in tumors containing or not containing Matrigel (data not shown).

By using labelled ^131^I-trastuzumab, we investigated if the presence of Matrigel could have influenced drug accumulation within the tumors. As shown in Figure 4c, a major amount of trastuzumab was quantified in tumors grown in the presence of Matrigel. Moreover, confocal microscopy analysis confirmed higher uptake of trastuzumab in Matrigel-mixed tumors than in tumors derived without Matrigel (Figure 4d,e), supporting the notion that increased drug delivery and efficacy is associated with collagen accumulation. These data suggested a clear influence of the cross-talk between tumor and ECM on trastuzumab efficacy.

## 4. Discussion

ECM3 is one group of BCs previously identified according to the expression of ECM genes [3], that is characterized by overexpression of a peculiar pattern of genes mainly encoding structural ECM molecules [4]. Our present analysis in cohorts of HER2-positive tumors identified ECM3 as a stable tumor partition also within this intrinsic subtype of BCs. Moreover, biological features of this subset of tumors are concordant with what we have previously described. Indeed, pathways related to EMT, hypoxia, tumor metastasis, TGFβ signaling pathway and adhesion to the matrix are enriched in these tumors, all features associated with tumor aggressiveness [29] that can explain the poor prognosis of patients with ECM3 tumors. Moreover, pathways related to stem cells (i.e., Wnt, Hedgehog pathways [30]) were found enriched in these tumors. This is in line with a described increase in stem cell markers when cells are exposed to collagen [31], and with the ability of BC cells with stem-like phenotypes to express an increased level of collagens [32].

ECM3 tumors were found associated with poor prognosis if untreated or treated with chemotherapy alone. It is likely that tumor microenvironment organization in ECM3 tumors contributes to resistance to chemotherapy. Several studies have shown that tumor microenvironment can contribute to resistance to chemotherapeutic agents through different mechanisms [2]. Resistance was described to be mainly associated with pro-survival signals derived from interaction between tumor cells and the matrix, or by the organization of the ECM that can limit blood flow and thus restrict diffusion of the drug into the tumor [33,34]. In this context, some genes belonging to the ECM3 pattern were described to be associated with resistance to tamoxifen in metastatic ER-positive BCs [35] and to FEC chemotherapy in neoadjuvant treated ER-negative BCs [36]. Moreover, in the HER2 subtype high SPARC expression, one of the leading genes of ECM3, other than being associated with poor prognosis, was found associated with low pCR rates in patients treated with anthracyclines and taxanes [37]. Furthermore, EMT and stem-like features, enriched in ECM3 tumors, have been largely associated with resistance to chemotherapy [38]. Thus, combination therapy targeting tumor and stromal characteristics also exploiting new approaches for drug delivery [39,40] could represent a new strategy to improve chemotherapy response in ECM3 tumors and requires further investigation.

Treatment with trastuzumab improves the prognosis of ECM3 patients and significantly increases the percentage of pCR. The high infiltration of ECM3 tumors by CD8+ T cells, described in a preclinical model to be relevant for trastuzumab activity [13] and that we previously found associated with trastuzumab benefit also in clinical samples [23], could be at the basis of the response. The presence of CD8+ cells in the ECM3 tumor microenvironment is in contrast with the poor prognosis of patients with this type of tumor, but the presence of immunosuppressive cells, like MDSC, described to be enriched in contact with tumor cells in ECM3 tumors [5], could suppress their antitumor activity. The association between ECM3 and immune metagenes in the NOAH dataset further supports an altered host response in ECM3 tumors, as observed in collagen-dense tumors [41]. The altered infiltration of immune cells in these tumors could be at the basis of trastuzumab efficacy. Higher expression of the T-cell surrogate LCK metagene and STAT1 metagene in ECM3 vs. non-ECM3 tumors who achieve pCR further supports this hypothesis.

Another possible explanation of the benefit of ECM3 tumors from trastuzumab derived from their stem cell-like features. Indeed, breast cancer stem cells rely on HER2 and are sensitive to trastuzumab [42]. In addition, the tumor microenvironment architecture derived from the production of ECM3 proteins could influence diffusion and accumulation of the drug in the tumor tissue as we found in Matrigel-mixed tumors in mice. These data seem to be in contrast with many studies illustrating the relevance of ECM density and composition in the ability of drugs to distribute and accumulate in the extravascular compartment of the tumor tissue [43]. However, trastuzumab has been shown to have a relatively high interstitial volume of distribution in solid tumor models due to its positive charge [44].

We are aware that the use of Matrigel to create an ECM-rich environment is not a perfect model of the ECM3 microenvironment, but our previous findings using this model, showing resistance to doxorubicin and low penetration of the drug in the tumor microenvironment [45], as observed in patients with ECM3 tumors, support our results. It cannot be excluded that the higher concentration of trastuzumab in these tumors derived from a different organization of the tumor microenvironment, characterized by a small nest of tumor cells separated by area of ECM, opposed to the tumor cell packed tumors derived from injection of tumor cells alone. The lack of Ki67 reduction by trastuzumab in xenografts is in line with data obtained in the MMTV/HER2 model [46] and in HER2-positive locally advanced BCs from patients treated with neoadjuvant trastuzumab [47], in which Ki67 is not modified by trastuzumab treatment. It is conceivable that tumor cell proliferation is inhibited by trastuzumab therapy, as observed in other models a few days after the start of the treatment [48,49]. Thus, it is likely that a reduction in proliferation rate occurs mainly at the beginning of the treatment, as suggested also by a strong reduction in Ki67 positivity in tumor tissue of HER2-positive BC patients of a window of opportunity trial treated with one cycle of trastuzumab [50].

The small number of patients and the heterogeneous group of tumors constitute the major limitation of the study. Further analysis of the predictive value of ECM3 is warranted. Moreover, from a clinical point of view, understanding whether the addition of ECM3 to the immune signatures increases the selection of patients who benefit from trastuzumab could represent an important issue for patient selection in the clinical practice.

## Figures and Tables

**Table 1:** Frequency of clinico-pathological characteristics of patients according to the extracellular matrix gene expression pattern (ECM3).

	EMC 29%		NKI 25%		FIRB 34%		GHEA 31%		NOAH 37%	
Variable	ECM3	Non-ECM3	ECM3	Non-ECM3	ECM3	Non-ECM3	ECM3	Non-ECM3	ECM3	Non-ECM3
	(*n* = 14)	(*n* = 35)	(*n* = 13)	(*n* = 39)	(*n* = 15)	(*n* = 29)	(*n* = 11)	(*n* = 25)	(*n* = 42)	(*n* = 72)
Estrogen receptor										
*Positive*	9 (64%)	24 (69%)	8 (62%)	25 (64%)	5 (33%)	16 (55%)	6 (55%)	12 (48%)	11 (26%)	16 (22%)
Tumor size										
*>T1*	NA	NA	7 (54%)	20 (51%)	13 (93%)	23 (79%)	5 (45%)	12 (48%)	NA	NA
Histological grade										
*III*	NA	NA	6 (46%)	24 (62%)	9 (69%)	19 (68%)	8 (73%)	20 (80%)	21 (50%)	40 (56%)
Lymph node status										
*Positive*	0	0	8 (62%)	18 (46%)	10 (71%)	19 (66%)	10 (91%)	21 (84%)	NA	NA

**Table 2:** Multivariate proportional hazard analyses of DFS.

	EMC		NKI		FIRB	
Variable	HR (95%CI)	*p*-Value	HR (95%CI)	*p*-Value	HR (95%CI)	*p*-Value
ECM3	5.50 (2.07–14.62)	0.0006	2.57 (1.06–6.19)	0.0361	2.29 (0.76–6.87)	0.1383
ER pos	0.58 (0.22–1.54)	0.2730	0.46 (0.20–1.09)	0.0781	1.09 (0.36–3.29)	0.8800

## Supplementary Materials

The following are available online at https://www.mdpi.com/2073-4409/9/2/434/s1: Figure S1: Association between ECM3 and PAM50 and TRAR. Figure S2: Association between ECM3 and immune metagenes. Figure S3: Body weight of mice treated or not with trastuzumab Table S1: Univariate proportional hazards analyses of DFS. Table S2: Multivariate proportional hazards analysis of DFS in NKI dataset. Table S3. Multivariate proportional hazards analyses of overall survival.
