# Supplementary material for: Extracellular Matrix Features Discriminate Aggressive HER2-Positive Breast Cancer Patients Who Benefit from Trastuzumab Treatment
Source: Cells. 2020 Feb 13;9(2):434. doi: 10.3390/cells9020434 (PMC7072535; doi:10.3390/cells9020434)
Supplement: Supplementary file 1 [file cells-09-00434-s001.zip › Figure S2.docx]

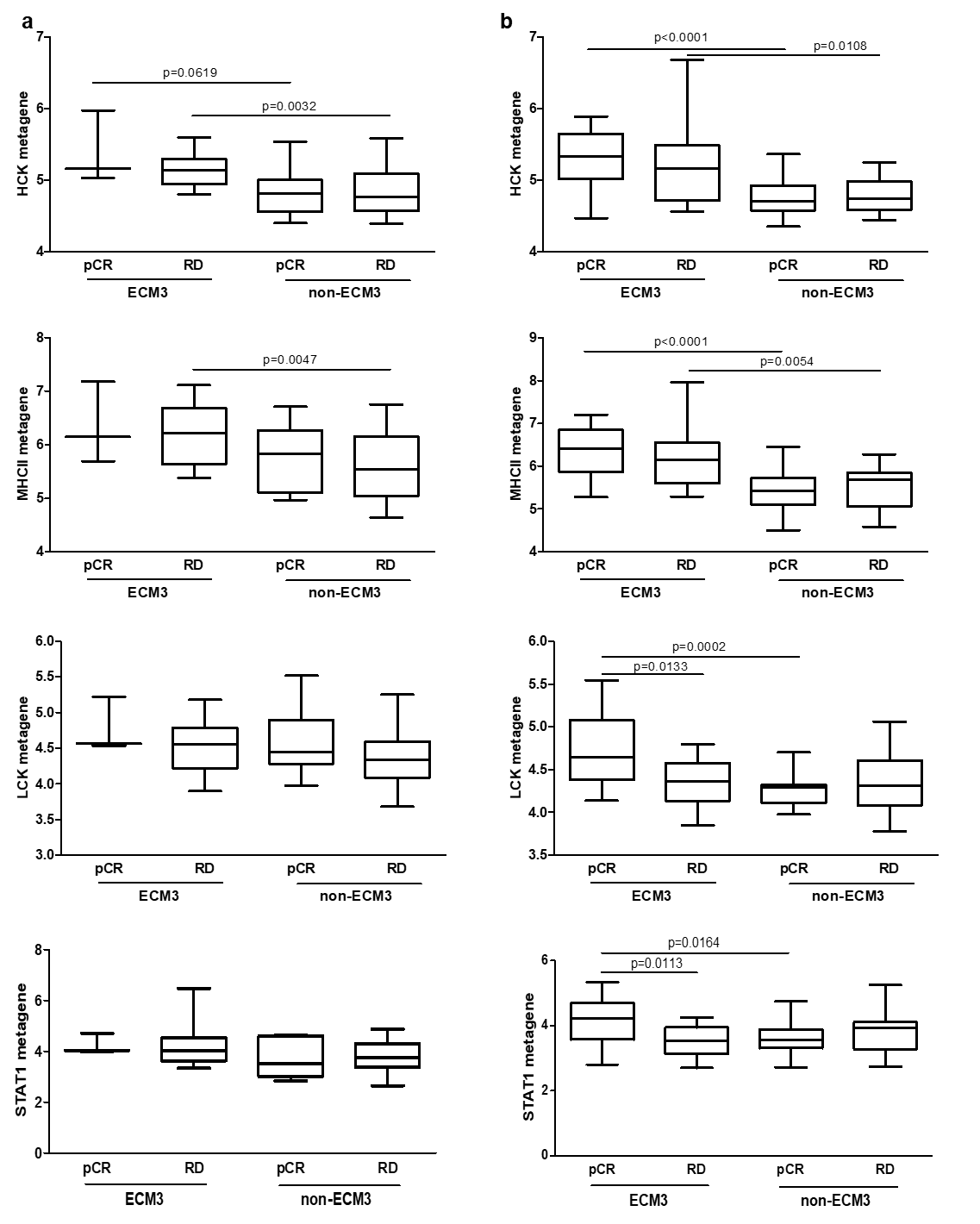


**Figure S2. Association between ECM3 and immune signatures. a-b)** Expression of immune metagenes in tumors of the NOAH dataset according to ECM classification and response to chemotherapy **(a)** or trastuzumab **(b).** p-values by unpaired t-test. pCR: pathological complete response, RD: residual disease.
