# Supplementary material for: Extracellular Matrix Features Discriminate Aggressive HER2-Positive Breast Cancer Patients Who Benefit from Trastuzumab Treatment
Source: Cells. 2020 Feb 13;9(2):434. doi: 10.3390/cells9020434 (PMC7072535; doi:10.3390/cells9020434)
Supplement: Supplementary file 1 [file cells-09-00434-s001.zip › Supplementary Table 1.docx]

**Supplementary Table 1. Univariate proportional hazards analyses of DFS.**

|  | **NKI** | | **EMC** | | **FIRB** | |
| --- | --- | --- | --- | --- | --- | --- |
| **Variable** | **HR (95%CI)** | **p-value** | **HR (95%CI)** | **p-value** | **HR (95%CI)** | **p-value** |
| ECM3 | 3.44 (1.24-9-50) | 0.0172 | 5.40 (2.04-14.3) | 0.0007 | 2.24 (0.78-6.39) | 0.1321 |
| ER pos | 0.78 (0.30-2.06) | 0.6227 | 0.60 (0.23-1.59) | 0.3098 | 0.86 (0.30-2.49) | 0.7850 |
| Size >T1 | 2.30 (0.85-6.24) | 0.1022 |  |  |  |  |
| Grade III | 1.19 (0.44-3.23) | 0.7290 |  |  | 0.97 (0.30-3.16) | 0.9619 |
| N pos |  |  |  |  | 1.48 (0.46-4.72) | 0.5119 |
