# Supplementary material for: Extracellular Matrix Features Discriminate Aggressive HER2-Positive Breast Cancer Patients Who Benefit from Trastuzumab Treatment
Source: Cells. 2020 Feb 13;9(2):434. doi: 10.3390/cells9020434 (PMC7072535; doi:10.3390/cells9020434)
Supplement: Supplementary file 1 [file cells-09-00434-s001.zip › Supplementary Table 2.docx]

**Table S2. Multivariate proportional hazards analysis of DFS.**

|  | **NKI** | |
| --- | --- | --- |
| **Variable** | **HR (95%CI)** | **p-value** |
| ECM3 | 2.61 (1.08-6.32) | 0.0338 |
| ER pos | 0.51 (0.21-1.22) | 0.1294 |
| Size >T1 | 2.31 (0.93-5.71) | 0.0710 |
