# Supplementary material for: Extracellular Matrix Features Discriminate Aggressive HER2-Positive Breast Cancer Patients Who Benefit from Trastuzumab Treatment
Source: Cells. 2020 Feb 13;9(2):434. doi: 10.3390/cells9020434 (PMC7072535; doi:10.3390/cells9020434)
Supplement: Supplementary file 1 [file cells-09-00434-s001.zip › Supplementary Table 3.docx]

**Supplementary Table 3. Multivariate proportional hazards analyses of overall survival.**

|  | **NKI** | | **FIRB** | |
| --- | --- | --- | --- | --- |
| **Variable** | **HR (95%CI)** | **p-value** | **HR (95%CI)** | **p-value** |
| ECM3 | 3.55 (1.30-10.23) | 0.0192 | 2.54 (0.81-8.02) | 0.1111 |
| ER pos | 0.92(0.33- 2.58) | 0.7562 | 0.87 (0.27-2.83) | 0.8214 |
