# Supplementary material for: Extracellular Matrix Features Discriminate Aggressive HER2-Positive Breast Cancer Patients Who Benefit from Trastuzumab Treatment
Source: Cells. 2020 Feb 13;9(2):434. doi: 10.3390/cells9020434 (PMC7072535; doi:10.3390/cells9020434)
Supplement: Supplementary file 1 [file cells-09-00434-s001.zip › Figure S1.docx]

**a b b**

**c d**

**Figure S1. Association between ECM3 and PAM50 and TRAR. a-b)** Percentage of HER2-enriched (HER2-E) and non-HER2-enriched (non-HER2-E) according to ECM classification in GHEA **(a)** and NOAH **(b)** cohorts. **c)** Percentage of TRAR-low and TRAR-high cases according to ECM classification in GHEA cohort. **d)** TRAR scores in tumors classified as ECM3 and non-ECM3 of the NOAH dataset.
